# Parental hesitancy and refusal toward routine childhood vaccination after the COVID-19 vaccination period: a cross-sectional study

**DOI:** 10.1186/s12875-026-03418-y

**Published:** 2026-06-12

**Authors:** Ozan Emre Eliaçık, Oğulcan Çöme, Tolga Günvar

**Affiliations:** Faculty of Medicine, Department of Family Medicine, Dokuz Eylul University, Mithatpasa Street, Balcova, Izmir, 35330 Turkey

**Keywords:** COVID-19, Vaccine hesitancy, Immunization programs, Parents, Child

## Abstract

**Background:**

The COVID-19 pandemic intensified public debates on vaccines, amplifying concerns about safety, side effects, rapid development, and institutional trust. These concerns may be associated with current attitudes toward routine childhood immunization. This study assessed parental intentions regarding routine childhood vaccination among parents whose children had previously completed routine immunizations.

**Methods:**

This cross-sectional analytical study included 320 parents of children aged 4–8 years who had completed routine childhood vaccinations and registered at Dokuz Eylul University Training Family Health Centers. Participants were selected by simple random sampling. Data were collected using a sociodemographic questionnaire, literature-based items on vaccine attitudes, and the Vaccine Hesitancy Scale. Analyses employed descriptive statistics, chi-square, t-tests, ANOVA and multinomial logistic regression.

**Results:**

Among parents whose children had completed routine immunizations before the pandemic, 9.7% reported refusal and 23.4% reported hesitancy if deciding today. In the multivariable model, lower educational status was independently associated with hesitancy, whereas lower income was associated with outright refusal. Previous acceptance of non-reimbursed childhood vaccines was inversely associated with both hesitancy and refusal. Side-effect perception, concerns about vaccine production and distrust in healthcare providers were independently associated with hesitancy, while vaccine effectiveness concerns and distrust in healthcare providers were independently associated with refusal.

**Conclusion:**

Hesitancy and refusal were observed among parents whose children had previously completed routine immunizations. Educational and socioeconomic factors, prior optional vaccine acceptance, vaccine-related concerns and healthcare provider trust were associated with current vaccination intention. Primary care-based, individualized vaccine communication is needed, while causal attitude changes cannot be inferred from this cross-sectional design.

**Supplementary Information:**

The online version contains supplementary material available at 10.1186/s12875-026-03418-y.

## Introduction

Vaccination is among the most effective public health interventions for preventing infectious diseases and premature mortality. The World Health Organization (WHO) defines vaccines as products that train the immune system to recognize and combat pathogens, thereby preventing vaccine-preventable diseases [1]. Population-level impacts are well documented: after widespread measles vaccination, global deaths fell from ~ 2.6 million annually in the pre-vaccine era to ~ 142,000 in 2018; poliomyelitis declined from ~ 350,000 cases annually in the late 1980s to only 33 cases in 2018 following intensified campaigns [2]. In Türkiye, routine childhood immunization—including Hepatitis B, BCG (Bacillus Calmette–Guérin), DTaP-IPV-Hib (diphtheria, tetanus, acellular pertussis, inactivated poliovirus, and Haemophilus influenzae type b), PCV (pneumococcal conjugate vaccine), MMR (measles, mumps, and rubella), IPV/OPV (inactivated poliovirus vaccine/oral poliovirus vaccine), Td (tetanus and diphtheria), Hepatitis A, and varicella—is provided free of charge through primary healthcare services. —has been systematically implemented and periodically updated by the Ministry of Health [3].

Despite these achievements, vaccine hesitancy threatens sustained high coverage. WHO’s Strategic Advisory Group of Experts (SAGE) defines hesitancy as delayed acceptance or refusal of vaccines despite service availability, shaped by complacency, convenience, and confidence; vaccine refusal denotes declining all vaccines by choice [4]. In 2019, WHO listed vaccine hesitancy among the top ten global health threats [5]. In Türkiye, families refusing vaccination increased from 5,091 in 2015 to 23,000 by 2017, mirroring global declines in confidence [6].

The COVID-19 (Coronavirus Disease 2019) pandemic intensified these dynamics. Rapid vaccine development, expedited authorization, intense media scrutiny, and an “infodemic” of misinformation and conspiracy theories eroded trust in vaccines and authorities in many settings [7]. Although discourse centered on COVID-19 vaccines, concerns raised during this period may have extended to routine childhood immunizations and may be associated with lower vaccine confidence and uptake. Several countries, including Türkiye, reported coverage decreases—particularly for MMR and DTaP-containing vaccines—accompanied by resurgences of measles and other vaccine-preventable diseases [8, 9]. Since the decline in vaccination rates affects not only unvaccinated individuals but also the overall immunity of the community, vaccine refusal should be regarded as an important public health issue concerning the entire society [10].

Understanding determinants of parental hesitancy—the key decision-makers for childhood vaccines—is therefore a priority. Determinants span contextual (media narratives, sociopolitical factors, access), individual/group (prior vaccination experiences, risk perceptions, trust in providers and institutions, health beliefs, information literacy), and vaccine-specific domains (perceived safety/efficacy, novelty, schedule complexity, cost, delivery). This study was guided by the WHO SAGE vaccine hesitancy framework, particularly the “3Cs” model and the broader vaccine hesitancy matrix. According to this framework, vaccine hesitancy is shaped by confidence, complacency, and convenience, as well as contextual, individual and group, and vaccine-specific influences. In the present study, variables related to trust in healthcare providers, perceived vaccine safety and effectiveness, concerns about vaccine production, religious considerations, prior vaccination behaviours, and sociodemographic characteristics were selected and interpreted within this framework [4].

Historically, organized resistance has periodically surged in response to mandates, safety scares, or contested evidence—from 19th-century smallpox laws to late-20th-century DTP controversies and the discredited MMR-autism claim—reinforcing narratives around autonomy and distrust of governmental and scientific authority [11–14]. In the 21st century, similar patterns have emerged around HPV and COVID-19 vaccines, with measurable spillover to pediatric schedules [15–17].

Evidence from Türkiye indicates a non-trivial and possibly growing burden of hesitancy. National and regional studies report declines in full immunization and increases in refusal or delay, with social media influence, prior delays, and lower trust in providers commonly implicated [8, 9, 18–20]. Hesitancy is not confined to the lay public; some primary care professionals report ambivalence, potentially affecting counseling and parental decisions [21]. Modeling suggests that, if trends persist, national coverage could fall below critical herd-immunity thresholds within five years, raising outbreak risks and potentially re-introducing previously rare infections [22].

Pandemic-era experiences may be associated with parental risk–benefit perceptions regarding vaccines. The heightened salience of adverse events (even if rare), ambiguity about variant-specific effectiveness, and polarized media frames can amplify perceived vaccine risks while attenuating perceived disease risks—particularly as pandemic fatigue reduces COVID-19’s perceived immediacy. Accelerated development timelines and evolving recommendations (e.g., boosters, variant-adapted formulations) may be interpreted as uncertainty rather than normal hallmarks of adaptive, evidence-based practice. In such contexts, personal vaccination behaviors (e.g., foregoing seasonal influenza vaccination), religious or cultural beliefs, and structural barriers (access, time costs) interact with misinformation to produce hesitancy or refusal.

Primary care is the front line for childhood immunization. Family Health Centers and Training Family Health Centers can monitor coverage, identify hesitant families, and deliver tailored communication and outreach. Yet the pandemic altered clinic workflows and risk-communication demands, possibly affecting the quality and frequency of vaccine counseling. Systematic, post-pandemic evidence linking parents’ COVID-19 vaccine experiences to current attitudes about routine childhood vaccines in primary care remains limited.

This study addresses that gap by focusing on parents of children aged 4–8 years who had previously completed the routine schedule. Consequently, the primary objective of this study is to investigate current parental behavioral intentions—specifically categorized as acceptance, hesitancy, and refusal—toward routine childhood vaccination following the COVID-19 vaccination period. Grounded in the WHO SAGE framework, this cross-sectional study systematically evaluates how specific socio-demographic characteristics, prior vaccination behaviors, and parent-reported perceptions are associated with these current vaccination decisions within a primary care setting.

## Methods

### Study design and settings

This cross-sectional analytical study assessed current parental attitudes and intentions regarding routine childhood vaccination after the COVID-19 vaccination period among parents of children aged 4–8 years who had previously completed routine childhood immunizations. The study population consisted of parents registered at the Dokuz Eylul University Training Family Health Centers in September, 2023 who had a child aged 4–8 years whose routine childhood immunizations were completed. Children’s completion of routine childhood immunizations before the COVID-19 pandemic was verified through the Turkish Ministry of Health data system. A sample of 320 parents was selected between September 1 and November 1, 2023, using a simple random sampling method. 

### Inclusion and exclusion criteria

Eligible participants were those aged 18 years and older who had a child aged 4–8 years with completed childhood immunizations. The only exclusion criterion was unwillingness to participate. 

### Sampling procedure

At the time of the study, there were 1890 eligible parents registered at the Dokuz Eylul University Training Family Health Centers who had at least one child aged 4–8 years with completed childhood immunizations. The sample size was calculated using OpenEpi. Based on a finite population of 1,890 eligible parents registered at Dokuz Eylul University Training Family Health Centers, a 95% confidence level, a 5% margin of error, and an expected prevalence of 50%, the minimum required sample size was calculated as 320 participants. Because the expected prevalence of parental vaccine hesitancy in this specific population was unknown, 50% was used as the most conservative assumption, as it yields the maximum required sample size. A list of all eligible parents was obtained from the registration records of the Training Family Health Centers. Participants were selected from this sampling frame using a computer-generated random number list. When selected parents could not be reached or declined participation, replacement participants were selected from the remaining eligible parents using the same random selection procedure. In total, 13 parents could not be reached and 3 declined participation; after replacement, 320 parents were enrolled in the study between September 1 and November 1, 2023. 

### Data collection instruments

Data were collected using a structured data collection form consisting of study-specific items and the Vaccine Hesitancy Scale (VHS). The study-specific items assessed sociodemographic characteristics, health-related factors, vaccination-related behaviours, current vaccination intention, and parent-reported perceived influences of COVID-19 vaccine debates. These items were developed by the authors based on the study objectives, the WHO SAGE vaccine hesitancy framework, and previous literature on parental vaccine hesitancy, COVID-19 vaccination attitudes, vaccine confidence, trust in healthcare providers, and vaccine-related misinformation [4, 16, 20, 23–30]. These additional items were not part of a previously validated scale and were analyzed as individual variables. An English translation of the full data collection form is provided as Supplementary File 1.

Vaccine hesitancy was measured using the Vaccine Hesitancy Scale (VHS) which was developed by the WHO SAGE Working Group and psychometrically evaluated by Shapiro et al. [31]. It is a five-point Likert instrument with nine items. For scoring, items in the “lack of confidence” subscale (items 1, 2, 3, 4, 6, 7, 8) were reverse-coded, while items in the “risk perception” subscale (items 5, 9) were summed directly. Higher total scores indicated greater vaccine hesitancy. There is no cut-off point for the Vaccine Hesitancy Scale. The Turkish validity and reliability study of this scale has been conducted by Önal et al. and the Cronbach’s alpha coefficient was found to be 0.856 [32]. 

### Study variables

The primary dependent variable was the parental decision on whether they would currently vaccinate their child with routine childhood vaccines which was assessed using a single categorical item: “If you were to decide today, would you have your child vaccinated with the routine childhood vaccines?” The response options were “Yes,” “Hesitant,” and “Would not vaccinate”. Independent variables included sociodemographic characteristics (age, sex, marital status, child’s sex, education level, income level, employment status, occupation), health-related factors (presence of chronic illness, regular medication use), and vaccination-related factors (COVID-19 vaccination status, history of COVID-19 infection, regular influenza vaccination, administration of optional vaccines not covered by the national program). Parent-reported perceived influences of COVID-19 vaccine debates were assessed using the item: “Which aspects of the COVID-19 vaccine debate have negatively influenced your overall perception of vaccines?” Multiple responses were allowed. The response options included side-effect concerns, vaccine effectiveness concerns, concerns about vaccine production processes, religious considerations, and trust in healthcare professionals/authorities. Vaccine hesitancy was additionally assessed using the Vaccine Hesitancy Scale. The selection and interpretation of independent variables were informed by the WHO SAGE vaccine hesitancy framework, including confidence-related, contextual, individual/group, vaccine-specific, and behavioural factors.

### Statistical analysis

Data were entered and analyzed using IBM SPSS Statistics version 27.0.1. Descriptive analyses were presented as counts and percentages for categorical variables and as means with standard deviations for continuous variables. The Kolmogorov–Smirnov test and skewness–kurtosis values were used to assess normality. For bivariate analyses, Pearson’s chi-square test or Fisher’s exact test were applied to categorical variables. For continuous variables, independent samples t-tests and one-way ANOVA were used for normally distributed data, while Mann–Whitney U and Kruskal–Wallis H tests were used for non-normally distributed data.

Multinomial logistic regression was used to identify factors associated with the three-category parental vaccination intention outcome, assessed using the single item: “If you were to decide today, would you have your child vaccinated with the routine childhood vaccines?” The response options were “Yes,” “Hesitant,” and “Would not vaccinate.” The “Yes” category was used as the reference outcome, and adjusted odds ratios (aORs) with 95% confidence intervals (CIs) were reported for “Hesitant” versus “Yes” and “Would not vaccinate” versus “Yes.” Candidate independent variables were selected based on theoretical relevance, clinical interpretability, and statistically significant associations in bivariate analyses. Before model fitting, cross-tabulations were examined to identify sparse cells and zero-event categories; occupation and regular seasonal influenza vaccination were excluded because they produced quasi-complete separation and unstable estimates. The final multivariable model included income level, education level, employment status, COVID-19 vaccination status, acceptance of non-reimbursed childhood vaccines, and parent-reported perceived influences of COVID-19 vaccine debates, including side-effect concerns, vaccine effectiveness concerns, concerns about vaccine production processes, religious concerns, and distrust in healthcare providers. Multicollinearity was assessed using variance inflation factors (VIFs). Model fit was evaluated using the − 2 log likelihood, likelihood-ratio chi-square test, Akaike information criterion (AIC), Bayesian information criterion (BIC), and Cox and Snell, Nagelkerke, and McFadden pseudo-R² statistics. Statistical significance was set at *p* < 0.05. 

### Ethical considerations

This study was conducted in accordance with the principles of the Declaration of Helsinki. Ethical approval was obtained from the Dokuz Eylul University Faculty of Medicine Non-Interventional Research Ethics Committee (Decision No: 2023/13–20, Date: 26.04.2023). Participants were provided with a detailed explanation of the study objectives, informed consent was obtained from all respondents prior to participation. Participation was voluntary, and confidentiality and anonymity were ensured throughout the study.

## Results

A total of 320 parents participated in the study, of whom 178 (55.6%) were female and 142 (44.4%) were male. The mean age of participants was 32.52 ± 5.53 years (range = 22–47). Most were married (*n* = 288, 90.0%) and employed (*n* = 231, 72.2%). Regarding education, 135 (42.2%) were university graduates, while 21 (6.6%) had no formal education. Nearly half of the participants (*n* = 157, 49.1%) reported household income equal to expenses, and 91 (28.4%) stated that their income exceeded expenses. 95 parents (29.7%) had at least one chronic disease, and 270 (84.4%) had received the COVID-19 vaccine. Children were almost equally distributed by gender (176 females, 55.0%; 144 males, 45.0%).

Regarding current routine childhood vaccination intention, 214 parents (66.9%) answered “Yes”, 75 (23.4%) answered “Hesitant” and 31 (9.7%) answered “Would not vaccinate” (Table 1).

**Table 1:** Parental decision on childhood vaccination by sociodemographic characteristics

Variable	Category	*n*	Yes 214 (66.9%)	Hesitant 75 (23.4%)	Would not vaccinate 31 (9.7%)	*p*
Parent’s Gender	Female	178	114 (64.0%)	44 (24.7%)	20 (11.2%)	0.416
	Male	142	100 (70.4%)	31 (21.8%)	11 (7.7%)	
Child’s Gender	Female	176	116 (65.9%)	40 (22.7%)	20 (11.4%)	0.529
	Male	144	98 (68.1%)	35 (24.3%)	11 (7.6%)	
Income Level	Income < Expenses	72	37 (51.4%)	18 (25.0%)	17 (23.6%)	< 0.001*
	Income = Expenses	157	102 (65.0%)	42 (26.8%)	13 (8.3%)	
	Income > Expenses	91	75 (82.4%)	15 (16.5%)	1 (1.1%)	
Employment Status	Employed	231	177 (76.6%)	40 (17.3%)	14 (6.1%)	< 0.001*
	Unemployed	89	37 (41.6%)	35 (39.3%)	17 (19.1%)	
Marital Status	Married	288	190 (66.0%)	69 (24.0%)	29 (10.1%)	0.572
	Single	32	24 (75.0%)	6 (18.8%)	2 (6.3%)	
Education Level	No formal education	21	6 (28.6%)	8 (38.1%)	7 (33.3%)	< 0.001*
	Primary school– High school	164	90 (54.9%)	55 (33.5%)	19 (11.6%)	
	University or higher	135	118 (87.4%)	12 (8.9%)	5 (3.7%)	
Occupation	Other	177	130 (73.4%)	34 (19.2%)	13 (7.3%)	< 0.001*
	Homemaker	86	35 (40.7%)	35 (40.7%)	16 (18.6%)	
	Teacher	27	19 (70.4%)	6 (22.2%)	2 (7.4%)	
	Health professional	30	30 (100.0%)	0 (0.0%)	0 (0.0%)	
Chronic Disease	Yes	95	65 (68.4%)	17 (17.9%)	13 (13.7%)	0.132
	No	225	149 (66.2%)	58 (25.8%)	18 (8.0%)	
COVID-19 Vaccination	Yes	270	200 (74.1%)	53 (19.6%)	17 (6.3%)	< 0.001*
	No	50	14 (28.0%)	22 (44.0%)	14 (28.0%)	
Side Effect after COVID-19 Vaccination	Yes	120	83 (69.2%)	25 (20.8%)	12 (10.0%)	0.062
	No	150	117 (78.0%)	28 (18.7%)	5 (3.3%)	
History of COVID-19	Yes	185	131 (70.8%)	36 (19.5%)	18 (9.7%)	0.137
	No	135	83 (61.5%)	39 (28.9%)	13 (9.6%)	
Regular Seasonal Influenza Vaccination	Yes	42	40 (95.2%)	2 (4.8%)	0 (0.0%)	< 0.001*
	No	278	174 (62.6%)	73 (26.3%)	31 (11.2%)	
Would you have your child receive non-reimbursed vaccines?	Yes	176	152 (86.4%)	22 (12.5%)	2 (1.1%)	< 0.001*
	No	144	62 (43.1%)	53 (36.8%)	29 (20.1%)	
“Which aspects of the COVID-19 vaccine debate have negatively influenced your overall perception of vaccines?”						
Influenced by Side Effect Perception	Yes	247	152 (61.5%)	68 (27.5%)	27 (10.9%)	< 0.001*
	No	73	62 (84.9%)	7 (9.6%)	4 (5.5%)	
Influenced by Vaccine Effectiveness	Yes	89	46 (51.7%)	28 (31.5%)	15 (16.9%)	< 0.001*
	No	231	168 (72.7%)	47 (20.3%)	16 (6.9%)	
Influenced by Vaccine Production Process	Yes	108	63 (58.3%)	31 (28.7%)	14 (13.0%)	0.064
	No	212	151 (71.2%)	44 (20.8%)	17 (8.0%)	
Influenced by Religious Reasons	Yes	21	4 (19.0%)	9 (42.9%)	8 (38.1%)	< 0.001*
	No	299	210 (70.2%)	66 (22.1%)	23 (7.7%)	
Influenced by Trust in Healthcare Providers	Yes	38	10 (26.3%)	16 (42.1%)	12 (31.6%)	< 0.001*
	No	282	204 (72.3%)	59 (20.9%)	19 (6.7%)	

### Sociodemographic and behavioral correlates

Parental vaccination attitudes varied significantly by education, income, and occupation (Table 1). Parents with university or higher education and those with income greater than expenses were more likely to report that they would vaccinate their children (*p* < 0.001). Conversely, participants with lower education or lower income demonstrated higher rates of vaccine hesitancy and refusal (*p* < 0.001). Occupational differences were also marked: all health professionals (*n* = 30) reported willingness to vaccinate, followed by teachers (70.4%) and other professions (73.4%), whereas homemakers (*n* = 86) exhibited the lowest acceptance rate (40.7%) and significantly higher hesitancy (*p* < 0.001). No significant associations were observed for parental gender (*p* = 0.416), child’s gender (*p* = 0.529), marital status (*p* = 0.572), or presence of chronic disease (*p* = 0.132).

Vaccination-related behaviours were significantly associated with current vaccination decision. Parents who had received COVID-19 vaccination (*n* = 270), influenza vaccination (*n* = 42), or who had accepted non-reimbursed vaccines for their children (*n* = 176) were significantly more likely to state they would vaccinate their child again (*p* < 0.001 for all comparisons). Among perceived influences of the COVID-19 vaccine debate, side-effect concerns (77.2%), doubts about efficacy (27.8%), religious beliefs (6.6%), and distrust in healthcare providers (11.9%) were each associated with lower likelihood of vaccinating (*p* < 0.001), whereas concerns about vaccine production processes were not (*p* = 0.064) (Table 1). 

### Vaccine hesitancy scale findings

Vaccine Hesitancy Scale (VHS) scores demonstrated significant variation across sociodemographic groups (Table 2). Lower income, unemployment, and lower education levels were associated with higher mean ranks for hesitancy (*p* < 0.001). Health professionals had the lowest VHS mean rank (58.4), and homemakers had the highest (212.1; *p* < 0.001). Parents vaccinated against COVID-19 or influenza, and those who had previously accepted non-reimbursed vaccines, exhibited significantly lower VHS scores (*p* < 0.001). In contrast, those who reported adverse effects after COVID-19 vaccination showed higher hesitancy (*p* < 0.001). History of COVID-19 infection did not significantly affect VHS scores (*p* = 0.44). Participants who indicated being influenced by concerns about side effects (*p* < 0.001), vaccine effectiveness (*p* = 0.015), religious beliefs (*p* < 0.001), or distrust in healthcare providers (*p* < 0.001) had significantly higher VHS scores. The highest mean scores were observed among those citing religious reasons (32.05 ± 6.30) and low trust in healthcare professionals (28.00 ± 7.15). Perceptions related to vaccine production were not significant (*p* = 0.93).

**Table 2:** Vaccine hesitancy scale scores by sociodemographic characteristics

Variable	Category	*n*	VHS Score (Mean ± SD /Mean Rank)	Lack of Confidence subscale	Risk subscale	*p*
Parent’s Gender	Female	178	22.46 ± 7.48	15.64 ± 6.17	6.81 ± 1.84	0.10
	Male	142	21.08 ± 7.33	14.53 ± 6.09	6.54 ± 1.89	
Child’s Gender	Female	176	22.04 ± 8.08	15.37 ± 6.70	6.67 ± 1.93	0.59
	Male	144	21.60 ± 6.57	14.88 ± 5.43	6.72 ± 1.80	
Income Level	Income < Expenses	72	215.53	213.31	200.74	< 0.001*
	Income = Expenses	157	164.20	163.32	167.85	
	Income > Expenses	91	110.57	113.86	115.98	
Employment Status	Employed	231	141.30	139.88	150.85	< 0.001*
	Unemployed	89	210.33	214.02	185.55	
Marital Status	Married	288	21.95 ± 7.47	15.29 ± 6.21	6.66 ± 1.85	0.42
	Single	32	20.84 ± 7.15	13.90 ± 5.52	6.93 ± 1.97	
Education Level	No formal education	21	220.90	223.31	195.07	< 0.001*
	Primary–High school	164	190.20	189.90	177.99	
	University or higher	135	115.02	115.01	133.88	
Occupation	Other	177	153.85	153.39	156.56	< 0.001*
	Homemaker	86	212.05	215.16	187.09	
	Teacher	27	153.39	147.59	175.02	
	Health professional	30	58.38	57.38	94.47	
Chronic Disease	Yes	95	21.82 ± 7.42	15.10 ± 6.07	6.71 ± 2.09	0.97
	No	225	21.85 ± 7.46	15.17 ± 6.20	6.68 ± 1.77	
COVID-19 Vaccination	Yes	270	20.56 ± 6.68	14.07 ± 5.48	6.49 ± 1.83	< 0.001*
	No	50	28.76 ± 7.52	21.00 ± 6.36	7.76 ± 1.72	
Side Effect after COVID-19 Vaccination	Yes	120	22.49 ± 7.04	15.49 ± 5.86	7.00 ± 1.85	< 0.001*
	No	150	19.02 ± 5.99	12.93 ± 4.89	6.09 ± 1.71	
History of COVID-19	Yes	185	22.12 ± 7.01	15.20 ± 5.91	6.92 ± 1.70	0.44
	No	135	21.46 ± 7.99	15.08 ± 6.50	6.37 ± 2.05	0.01* (risk subscale)
Regular Seasonal Influenza Vaccination	Yes	42	16.80 ± 4.78	11.21 ± 3.82	5.59 ± 1.71	< 0.001*
	No	278	22.60 ± 7.47	15.74 ± 6.23	6.85 ± 1.84	
Would you have your child receive non-reimbursed vaccines?	Yes	176	17.94 ± 5.11	11.85 ± 4.01	6.09 ± 1.74	< 0.001*
	No	144	26.61 ± 7.05	19.18 ± 5.92	7.43 ± 1.75	
“Which aspects of the COVID-19 vaccine debate have negatively influenced your overall perception of vaccines?”						
Influenced by Side Effect Perception	Yes	247	22.72 ± 7.34	15.77 ± 6.16	6.94 ± 1.82	< 0.001*
	No	73	18.90 ± 7.04	13.05 ± 5.93	5.85 ± 1.78	
Influenced by Vaccine Effectiveness	Yes	89	23.61 ± 8.22	16.63 ± 6.88	6.98 ± 1.92	0.015*
	No	231	21.17 ± 7.01	14.58 ± 5.77	6.58 ± 1.84	
Influenced by Vaccine Production Process	Yes	108	22.82 ± 7.37	15.92 ± 6.17	6.91 ± 1.75	0.93
	No	212	21.35 ± 7.44	14.76 ± 6.13	6.58 ± 1.92	
Influenced by Religious Reasons	Yes	21	32.05 ± 6.30	23.76 ± 5.45	8.29 ± 1.65	< 0.001*
	No	299	21.13 ± 6.98	14.55 ± 5.74	6.58 ± 1.83	
Influenced by Trust in Healthcare Providers	Yes	38	28.00 ± 7.15	20.05 ± 6.21	7.95 ± 1.66	< 0.001*
	No	282	21.02 ± 7.09	14.49 ± 5.85	6.52 ± 1.83	

In the present sample, the internal consistency of the Vaccine Hesitancy Scale was assessed using Cronbach’s alpha. The Cronbach’s alpha coefficient was 0.929 for the total scale, 0.936 for the lack of confidence subscale, and 0.786 for the risk perception subscale. 

### Multivariable analysis

A multinomial logistic regression model was constructed to identify factors independently associated with parents’ current vaccination decision. For the outcome variable based on the question,”If you were to decide today, would you have your child vaccinated with the routine childhood vaccines?”, the response category “Yes” was used as the reference outcome, allowing separate comparisons for “Hesitant” versus “Yes” and “Would not vaccinate” versus “Yes.” Therefore, adjusted odds ratios greater than 1 indicate higher odds of hesitancy or refusal compared with willingness to vaccinate, whereas adjusted odds ratios below 1 indicate lower odds of hesitancy or refusal relative to the “Yes” group.

In preliminary analyses, occupation and regular seasonal influenza vaccination produced unstable estimates because of zero-event categories; therefore, these variables were not retained in the final multivariable model.

For the comparison of “Hesitant” versus “Yes,” educational attainment remained independently associated with hesitancy. Compared with parents with university or higher education, parents with no formal education had higher odds of being hesitant rather than willing to vaccinate (aOR = 6.81, 95% CI: 1.34–34.58; *p* = 0.021). Similarly, parents with primary to high-school education had higher odds of hesitancy compared with those with university or higher education (aOR = 4.84, 95% CI: 1.79–13.08; *p* = 0.002). Acceptance of non-reimbursed vaccines was inversely associated with hesitancy; parents who had accepted non-reimbursed childhood vaccines had lower odds of being hesitant compared with those who had not accepted such vaccines (aOR = 0.34, 95% CI: 0.17–0.70; *p* = 0.003). Employment status was not independently associated with hesitancy at the conventional significance level (aOR = 2.14, 95% CI: 0.96–4.79; *p* = 0.063) (Table 3).

**Table 3:** Multinomial logistic regression analysis for parental responses to the question “If you were to decide today, would you have your child vaccinated with the routine childhood vaccines?”

Variable	Hesitant vs. Yes aOR	95% CI	*p*	Would not vaccinate vs. Yes aOR	95% CI	*p*
Income level						
Income < expenses	0.79	0.27–2.31	0.666	35.80	2.58–496.99	0.008*
Income = expenses	1.29	0.52–3.22	0.586	15.58	1.31–185.09	0.030*
Income > expenses	Reference			Reference		
Education level						
No formal education	6.81	1.34–34.58	0.021*	1.35	0.16–11.69	0.788
Primary–high school	4.84	1.79–13.08	0.002*	0.60	0.12–3.03	0.539
University or higher	Reference			Reference		
Employment status						
Unemployed	2.14	0.96–4.79	0.063	2.97	0.94–9.41	0.064
Employed	Reference			Reference		
COVID-19 vaccination status						
Vaccinated	0.81	0.32–2.02	0.648	0.59	0.19–1.87	0.371
Unvaccinated	Reference			Reference		
Acceptance of non-reimbursed vaccines						
Yes	0.34	0.17–0.70	0.003*	0.04	0.01–0.21	< 0.001*
No	Reference			Reference		
Parent-reported COVID-19 vaccine debate concerns						
Side-effect perception						
Yes	4.90	1.85–12.96	0.001*	2.89	0.75–11.17	0.124
No	Reference			Reference		
Vaccine effectiveness concerns						
Yes	2.02	0.98–4.17	0.057	4.25	1.49–12.10	0.007*
No	Reference			Reference		
Vaccine production-process concerns						
Yes	3.40	1.52–7.63	0.003*	2.57	0.83–7.91	0.101
No	Reference			Reference		
Religious reasons						
Yes	2.11	0.48–9.36	0.326	3.14	0.55–17.82	0.196
No	Reference			Reference		
Distrust in healthcare providers						
Yes	3.29	1.10–9.82	0.033*	7.15	1.70–30.00	0.007*
No	Reference			Reference		

The outcome reference category was “Yes.” Adjusted odds ratios are presented for “Hesitant” versus “Yes” and “Would not vaccinate” versus “Yes”

*aOR* adjusted odds ratio, *CI* confidence interval

Among parent-reported concerns related to COVID-19 vaccine debates, side-effect perception was independently associated with hesitancy. Parents who reported that side-effect-related discussions had negatively influenced their overall perception of vaccines had higher odds of being hesitant rather than willing to vaccinate (aOR = 4.90, 95% CI: 1.85–12.96; *p* = 0.001). Concerns about vaccine production processes were also independently associated with hesitancy (aOR = 3.40, 95% CI: 1.52–7.63; *p* = 0.003). In addition, distrust in healthcare providers was associated with higher odds of hesitancy (aOR = 3.29, 95% CI: 1.10–9.82; *p* = 0.033). Vaccine effectiveness concerns were not independently associated with hesitancy at the conventional significance level, although the estimate suggested a possible positive association (aOR = 2.02, 95% CI: 0.98–4.17; *p* = 0.057). Religious reasons were not independently associated with hesitancy in the adjusted model (aOR = 2.11, 95% CI: 0.48–9.36; *p* = 0.326). Income level and COVID-19 vaccination status were also not independently associated with hesitancy in the final model.

For the comparison of “Would not vaccinate” versus “Yes,” income level was independently associated with outright refusal. Compared with parents whose income exceeded expenses, parents whose income was lower than expenses had markedly higher odds of refusing routine childhood vaccination (aOR = 35.80, 95% CI: 2.58–496.99; *p* = 0.008). Parents whose income was equal to expenses also had higher odds of refusal compared with those whose income exceeded expenses (aOR = 15.58, 95% CI: 1.31–185.09; *p* = 0.030). However, the wide confidence intervals indicate limited precision, and these estimates should be interpreted cautiously (Table 3).

Acceptance of non-reimbursed vaccines remained strongly inversely associated with refusal. Parents who had accepted non-reimbursed childhood vaccines had substantially lower odds of refusing routine childhood vaccination compared with those who had not accepted such vaccines (aOR = 0.04, 95% CI: 0.01–0.21; *p* < 0.001). COVID-19 vaccination status was not independently associated with refusal after adjustment for sociodemographic factors, vaccination-related behaviours, and parent-reported vaccine debate concerns (aOR = 0.59, 95% CI: 0.19–1.87; *p* = 0.371).

Among parent-reported concerns, vaccine effectiveness concerns and distrust in healthcare providers were independently associated with refusal. Parents who reported that discussions about vaccine effectiveness had negatively influenced their overall perception of vaccines had higher odds of refusing routine childhood vaccination (aOR = 4.25, 95% CI: 1.49–12.10; *p* = 0.007). Distrust in healthcare providers was also strongly associated with refusal (aOR = 7.15, 95% CI: 1.70–30.00; *p* = 0.007). Side-effect perception, concerns about vaccine production processes, and religious reasons were not independently associated with refusal in the final adjusted model.

The final model was statistically significant based on the likelihood-ratio test (χ² = 174.46, df = 24, *p* < 0.001). Model fit indices were as follows: −2 log likelihood = 360.10, Cox and Snell R² = 0.420, Nagelkerke R² = 0.518, McFadden R² = 0.326, AIC = 412.10, and BIC = 510.08. All variance inflation factor values were below 2.1, indicating no evidence of problematic multicollinearity. Detailed model fit and multicollinearity diagnostics are provided in Supplementary Tables 1 and 2.

## Discussion

This cross-sectional study assessed current parental hesitancy and refusal toward routine childhood vaccination after the COVID-19 vaccination period among parents whose children had completed routine immunizations before the pandemic. Unlike most previous studies that assessed vaccine perceptions after the pandemic, the present study shows that current hesitancy and refusal may be observed even among families with documented completion of routine childhood vaccination before the pandemic. 

### Principal findings

The study revealed that even among families with previously complete vaccination histories, 9.7% of parents reported that they would not vaccinate their child with routine childhood vaccines if deciding today, and 23.4% reported hesitancy. These rates are consistent with national and international findings showing increased parental uncertainty following the COVID-19 vaccination campaign [19, 33]. Meta-analytical evidence indicates global childhood-vaccine hesitancy prevalence ranging between 3% and 2%, depnding on income level [33]. The observed rates thus fall within expected ranges but are noteworthy given the participants’ prior adherence to immunization schedules. The observed associations suggest that concerns commonly discussed during the COVID-19 vaccination period coexist with current hesitancy and refusal toward routine childhood vaccines. 

### Sociodemographic context

Women constituted 55.6% of the sample, mirroring the predominance of mothers in child-health–related research and decision-making [34–36]. The educational profile—over 40% withuniversity-level education—exceeded national averages [37], likely reflecting the higher socioeconomic status of the study area and the greater willingness of more educated individuals to participate in scientific studies. This context may partly explain the moderate rather than extreme hesitancy levels, as education and income are known protective factors against misinformation. 

### Influence of COVID-19 vaccination experience

Among participants, 84.4% had received the COVID-19 vaccine. Vaccine-hesitancy scores were significantly higher in unvaccinated individuals, and the proportion responding “Yes, I would vaccinate my child again” was markedly lower in bivariate analyses. These results are consistent with previous studies reporting an association between COVID-19 vaccine refusal and childhood-vaccine hesitancy [23, 24]. However, COVID-19 vaccination status was not independently associated with hesitancy or refusal in the final multivariable model after adjustment for sociodemographic factors, vaccination-related behaviours, and parent-reported concerns related to COVID-19 vaccine debates. This suggests that the relationship between parental COVID-19 vaccination status and childhood vaccination intention may be partly explained by broader concerns about vaccine safety, vaccine effectiveness, and trust in healthcare providers.

Participants reporting post COVID-19 vaccination side effects exhibited significantly higher vaccine hesitancy scores. Negative personal experiences may be associated with heightened perceived risk and lower vaccine confidence. Comparable findings by Bani Hani et al. demonstrated that mothers with prolonged adverse symptoms after COVID-19 vaccination were more likely to reject new childhood vaccines [38]. Nevertheless, no significant difference emerged regarding the decision to vaccinate children in the multivariable model, implying that although adverse experiences may increase concern, they may not necessarily translate into outright refusal.

An alternative explanation should also be considered. Parents who did not receive COVID-19 vaccination may have had lower vaccine confidence before the pandemic, rather than developing lower confidence as a result of pandemic-related debates. Similarly, previous acceptance of non-reimbursed childhood vaccines may not represent an independent protective factor by itself, but may reflect pre-existing vaccine confidence, higher socioeconomic status, better access to health information, greater trust in physicians, or greater ability to pay for optional vaccines. 

### Past illness and preventive behavior

Having experienced COVID-19 did not significantly affect overall hesitancy scores, though individuals who had been infected displayed higher “risk perception” subscale scores. This finding parallels Akbulut et al., indicating that infection itself does not shift overall attitudes but may sensitize individuals to potential risks [24]. Regular seasonal influenza vaccination was associated with lower VHS scores and greater willingness to vaccinate children in bivariate analyses, consistent with previous findings suggesting that prior positive vaccination experiences may reinforce vaccine confidence [34, 39]. However, because no regular influenza vaccine recipients reported outright refusal for their child, this variable produced a zero-event category and was not retained in the final multivariable model. 

### Socioeconomic and occupational correlates

Working participants demonstrated lower vaccine hesitancy scores and higher acceptance compared with non-working individuals, though this association lost significance in multivariable analysis. Lower education and income levels among the unemployed likely confounded this relationship. Consistent with prior literature, economic stability and employment correlate with better health literacy and vaccine acceptance [40].

Income level was associated with both VHS scores and current vaccination intention in bivariate analyses. Parents whose income was lower than their expenses had higher VHS scores and lower willingness to vaccinate their children compared with parents whose income exceeded expenses. In the final multivariable model, however, income level was not independently associated with hesitancy, but it was associated with outright refusal. Parents whose income was lower than expenses and those whose income was equal to expenses had higher odds of refusing routine childhood vaccination compared with parents whose income exceeded expenses. Nevertheless, the wide confidence intervals indicate limited precision, and this finding should be interpreted cautiously. This pattern mirrors national and international data showing that socioeconomic disadvantage is linked to mistrust and lower vaccine uptake [19, 34].

Education level was associated with VHS scores in bivariate analyses, with lower educational level corresponding to higher vaccine hesitancy scores. Education status was independently associated with hesitancy in the final multivariable model. Compared with parents with university or higher education, those with no formal education and those with primary to high-school education had higher odds of being hesitant rather than willing to vaccinate. Numerous studies corroborate the inverse relationship between education and vaccine hesitancy [34, 40, 41]. Higher education may support vaccine confidence by improving health literacy, critical appraisal of online information, and reliance on evidence-based sources. However, education was not independently associated with outright vaccine refusal in the adjusted model.

In descriptive analyses, healthcare professionals exhibited the lowest vaccine hesitancy and highest acceptance rates among occupational groups, while homemakers scored highest on hesitancy. Similar results have been documented internationally [40]. However, occupation was not included in the final multivariable model because all healthcare professionals reported willingness to vaccinate, resulting in a zero-event category and unstable estimates. Therefore, occupational differences should be interpreted as descriptive patterns. Familiarity with scientific information and daily engagement with immunization practices may buffer health professionals against misinformation. However, the possibility of social-desirability bias—responding in line with professional expectations—should also be considered. 

### Determinants of perception change during the pandemic

When participants were asked which pandemic-related discussions most influenced their perception of vaccines, the most frequently cited concerns were side effects (77.2%), vaccine-production processes (33.8%), efficacy (27.8%), healthcare-worker reliability (11.9%), and religious reasons (6.6%). In bivariate analyses, concerns about side effects, vaccine effectiveness, religious reasons, and distrust in healthcare providers were associated with lower willingness to vaccinate and higher VHS scores. These associations parallel findings by Moucheraud et al., Pervane et al., and Grills & Wagner, who observed substantial post-pandemic increases in parental fear of adverse effects and diminished confidence in vaccine safety and effectiveness [25–27].

In the final multivariable model, these concerns showed different patterns for hesitancy and outright refusal. Side-effect perception, concerns about vaccine production processes, and distrust in healthcare providers were independently associated with hesitancy. This suggests that uncertainty about vaccine safety and production may contribute particularly to indecision rather than complete rejection. Such concerns may reflect the heightened public attention to adverse events, rapid vaccine development, and changing recommendations during the COVID-19 vaccination period.

Religious reasons were associated with lower willingness to vaccinate and higher VHS scores in bivariate analyses, but they were not independently associated with hesitancy or refusal in the final adjusted model. Such beliefs are often deeply rooted and resistant to change, as highlighted in studies among Muslim populations linking vaccine hesitancy to concerns about non-halal ingredients or fatalistic views of illness [28, 29]. Targeted engagement with faith leaders and culturally sensitive communication may therefore be required to address this subgroup.

Finally, vaccine effectiveness concerns and distrust in healthcare providers remained independently associated with higher odds of vaccine refusal. Moucheraud et al. demonstrated that trust in healthcare workers independently predicts child immunization adherence [25]. The 2023 UNICEF (United Nations Children’s Fund) State of the World’s Children Report similarly noted that contradictory expert opinions and rapidly changing official guidance during COVID-19 eroded confidence in information sources [30]. Notably, distrust in healthcare providers was associated with both hesitancy and refusal, emphasizing the central role of trust in primary care-based vaccine communication. 

### Implications

These findings support the need for primary care-based vaccine communication that directly addresses the main factors associated with hesitancy and refusal, including lower socioeconomic and educational background, concerns about side effects, vaccine production processes, vaccine effectiveness, and trust in healthcare providers. Communication should be clear, individualized, and non-judgmental, allowing parents to express concerns while receiving evidence-based information. The association of distrust in healthcare providers with both hesitancy and refusal underscores the importance of continuity of care and a strong patient–physician relationship. Families who have not accepted non-reimbursed childhood vaccines may also represent an important group for targeted counselling. 

### Limitations

This study has several limitations. First, its cross-sectional design precludes causal inference; since pre-pandemic parental attitudes were not measured prospectively, findings reflect current vaccination intentions rather than longitudinal attitude changes over time. Second, the primary outcome captures behavioral intention at a single time point rather than subsequently observed behavior. While children’s routine immunizations and parental records were verified via the Turkish Ministry of Health data system, parent-reported perceptions regarding COVID-19 vaccine debates may still be subject to recall or attribution bias. Third, because the study was conducted in a single university-affiliated setting with a relatively highly educated sample, findings may not be fully generalizable to the broader population in Türkiye, particularly families with lower socioeconomic backgrounds or different healthcare access. Fourth, social-desirability bias may have caused underreporting of hesitancy, particularly among healthcare professionals due to professional norms. Similarly, the exclusion of parents unwilling to participate may have introduced selection bias if highly hesitant parents opted out. Finally, although variables causing quasi-complete separation were excluded to ensure model stability, the small number of parents in the refusal category resulted in wide confidence intervals for some estimates (e.g., income), requiring cautious interpretation.

## Conclusion

In this cross-sectional study, current hesitancy and refusal toward routine childhood vaccination were observed among parents whose children had completed routine immunizations before the COVID-19 pandemic. Lower educational status was independently associated with hesitancy, whereas lower income was associated with outright refusal, although the latter estimates should be interpreted cautiously because of wide confidence intervals. Previous acceptance of non-reimbursed childhood vaccines was inversely associated with both hesitancy and refusal. Parent-reported concerns about side effects, vaccine production processes and distrust in healthcare providers were associated with higher vaccine hesitancy, with distrust in healthcare providers emerging as relevant to both hesitancy and refusal. Concerns about vaccine effectiveness were associated with higher vaccine refusal. These findings suggest that parental vaccination decisions are closely linked to contemporary safety anxieties, efficacy perceptions, and institutional trust, while acknowledging that causal changes in attitudes over time cannot be inferred from the present design. 

## Supplementary Information

Supplementary Material 1.

## Data Availability

The data supporting the findings of this study are available upon reasonable request from the corresponding author. Due to ethical considerations and participant confidentiality, data cannot be shared publicly.

## Abbreviations

BCG: Bacillus Calmette–Guérin
CI: Confidence Interval
COVID-19: Coronavirus Disease 2019
DTaP: Diphtheria, Tetanus, and Acellular Pertussis vaccine
DTP: Diphtheria, Tetanus, and Pertussis vaccine
Hib: Haemophilus influenzae type b
HPV: Human Papillomavirus
IPV: Inactivated Poliovirus Vaccine
MMR: Measles, Mumps, and Rubella vaccine
OPV: Oral Poliovirus Vaccine
OR: Odds Ratio
PCV: Pneumococcal Conjugate Vaccine
SAGE: Strategic Advisory Group of Experts
SD: Standard Deviation
Td: Tetanus and Diphtheria vaccine
TFMRA: Taiwan Family Medicine Research Award
UNICEF: United Nations Children’s Fund
VHS: Vaccine Hesitancy Scale
WHO: World Health Organization
