# Supplementary material for: Parental hesitancy and refusal toward routine childhood vaccination after the COVID-19 vaccination period: a cross-sectional study
Source: BMC Prim Care. 2026 Jun 12;27:310. doi: 10.1186/s12875-026-03418-y (PMC13495287; doi:10.1186/s12875-026-03418-y)
Supplement: Supplementary file 1 [file 12875_2026_3418_MOESM1_ESM.docx]

**Supplementary File 1**

**English Translation of the Data Collection Form**

*Parental Hesitancy and Refusal Toward Routine Childhood Vaccination After the COVID-19 Vaccination Period: A Cross-Sectional Study*

**1) Age:**

……..

**2) Sex:**

□ Female	□ Male

**3) Marital status:**

□ Married	□ Not married

**4) What is your child’s sex?**

□ Girl	□ Boy

**5) Income level:**

□ My income is lower than my expenses	□ My income is equal to my expenses	□ My income is higher than my expenses

**6) Educational status:**

□ Literate	□ Primary or secondary school graduate	□ High school graduate	□ University or higher

**7) Employment status:**

□ Employed	□ Unemployed

**8) Occupation:**

……..

**9) Do you have any disease for which you regularly receive or should receive treatment?**

□ Yes	□ No

**10) Do you regularly use any medication for any reason?**

□ Yes	□ No

**11) Have you received a COVID-19 vaccine?**

□ Yes	□ No

**12) If you answered “Yes” to Question 11, please answer this question: Did you experience any side effect after receiving the COVID-19 vaccine?**

□ Yes	□ No

**13) Have you had COVID-19?**

□ Yes	□ No

**14) Do you regularly receive the influenza vaccine?**

□ Yes	□ No

**15) Would you have your child receive vaccines not reimbursed by the government? (e.g., meningococcal, rotavirus)**

□ Yes	□ No

**16) Which aspects of the COVID-19 vaccine debate that you witnessed negatively influenced your overall perception of vaccines? (Multiple responses may be selected.)**

□ Side effects	□ Effectiveness of vaccines	□ Trust in the vaccine production process	□ Religious reasons	□ Reliability of healthcare workers	□ Other: ……………………

**17) If you were to decide today, would you have your child vaccinated with routine childhood vaccines?**

□ Yes	□ Hesitant	□ I would not

**Vaccine Hesitancy Scale Items**

Please indicate your opinion about the following statements by marking the appropriate response option.

*Response options: 1 = Strongly disagree; 2 = Disagree; 3 = Undecided; 4 = Agree; 5 = Strongly agree.*

| **Statement** | **1** | **2** | **3** | **4** | **5** |
| --- | --- | --- | --- | --- | --- |
| Childhood vaccines are important for my child’s health. | □ | □ | □ | □ | □ |
| Childhood vaccines are effective. | □ | □ | □ | □ | □ |
| Having my child vaccinated is important for the health of others in my community. | □ | □ | □ | □ | □ |
| All childhood vaccines provided by the government in our society are beneficial. | □ | □ | □ | □ | □ |
| New vaccines carry more risks than older vaccines. | □ | □ | □ | □ | □ |
| The information I receive about vaccines from the vaccination program is reliable. | □ | □ | □ | □ | □ |
| Vaccination is a good way to protect my children from diseases. | □ | □ | □ | □ | □ |
| I generally do what my doctor or allied healthcare personnel (e.g., midwife, nurse) recommend about vaccines for my children. | □ | □ | □ | □ | □ |
| I am concerned about serious side effects of vaccines. | □ | □ | □ | □ | □ |

*Note: This supplementary file is an English translation of the data collection form used in the study.*
